# Comprehensive evaluation of the development of traditional Chinese medicine industry in Shaanxi province based on PMC index model

**DOI:** 10.3389/fpubh.2025.1500603

**Published:** 2025-02-24

**Authors:** Xiaoying Zhu, Shuzhi Lin, Wei Liu, Qian Liu, Lin Yin, Bianling Feng

**Affiliations:** Department of Pharmacy Administration, School of Pharmacy, Xi’an Jiaotong University, Xi’an, Shaanxi, China

**Keywords:** traditional Chinese medicine policies, traditional Chinese medicine (TCM) industry development, PMC-index model, principal component analysis (PCA), comprehensive evaluation

## Abstract

**Background:**

The high-quality development of the traditional Chinese medicine (TCM) industry is dependent on supportive policies and requires higher levels of coordination and integration. National and local government policies must coordinate the integrated development of the TCM industry under modern governance principles. However, the policy structure and its impact on the development of the TCM industry have not been thoroughly explored from the perspectives of policy design and empirical evidence.

**Methods:**

A TCMIDPs evaluation system was established using the PMC-Index model to quantitatively analyze TCMIDPs in China and Shaanxi Province. Additionally, principal component analysis (PCA) was used to comprehensively evaluate 51 enterprises in Shaanxi Province.

**Results:**

The policy analysis shows that the average PMC index of the 31 TCM industry development policies is 5.080, indicating that the areas and functions covered by these policies need improvement. The comprehensive evaluation reveals that pharmaceutical enterprises in Shaanxi Province still exhibit unbalanced development.

**Conclusion:**

Future TCM industry policies should focus on long-term industrial development, incorporating comprehensive perspectives. Emphasis should be placed on strengthening enterprise R&D, accelerating pharmaceutical innovation, and promoting the holistic development of pharmaceutical enterprises.

## 1 Introduction

In China, traditional Chinese medicine (TCM) has a history spanning thousands of years and is a crucial part of traditional Chinese culture (1). Evidence suggests that TCM has played a significant role in disease treatment, preventive healthcare, and public health (2–4). In 2019, the World Health Organization (WHO) included TCM diagnostics in the 11th revision of its International Classification of Diseases (ICD-11), allowing for statistical data that encompasses information beyond western medicine, with long-term implications for TCM (5, 6). Countries including the United States, Canada, and Australia have established formal education and certification systems for TCM (7–9). Additionally, several countries have established dedicated TCM research institutions and treatment centers (10), reflecting the growing global interest and recognition of TCM.

The TCM industry is a major component of China’s economy (11), covering sectors such as the cultivation, processing, and sale of medicinal herbs, as well as the research and production of TCM formulations (12). The Chinese government has concurrently implemented various policy measures (13, 14) and allocated substantial financial resources to support innovation and technological advancements in TCM (15, 16), aiming to promote exports and regional economic development. Despite these efforts, the TCM industry faces several challenges, including insufficient standardization and regulation of medicinal material production, limited innovation and research capacity for TCM products, small-scale enterprises, and weak brand influence (15, 17, 18). These challenges undermine the global competitiveness of TCM products, hindering the industry’s overall growth. Furthermore, the impact of government policies on the TCM industry and its current development requires further investigation.

Policy evaluation has become crucial in addressing these challenges. Sustainable, high-quality development of the TCM sector requires strong policy support and improved coordination across levels. Industrial promotion policies at national and local levels should integrate TCM industry and service development within modern governance frameworks. Existing studies indicate that the PMC-Index model provides a comprehensive approach to evaluating industrial promotion policies, offering valuable insights and methodologies. This model has been widely used to assess policies in various fields, including economics, employment, and healthcare (19–21). Compared to other evaluation methods, it integrates qualitative and quantitative analysis effectively, providing a more holistic and objective assessment (22).

The Shaanxi region is a crucial production area for authentic medicinal materials in China and exhibits regional characteristics in developing the TCM industry (23). Therefore, this study aims to explore the TCM industry’s development from this perspective. Considering that existing research has yet to analyze the policy structure and its impact on TCM industry development from the perspectives of policy design and empirical evidence, this research uses the PMC-Index model to construct a reasonable evaluation index system for TCM industry development policies (TCMIDPs), analyzing the quality of TCMIDPs from a policy perspective. Simultaneously, using principal component analysis (PCA), it investigates the main issues of the TCM industry in Shaanxi by collecting data from 51 TCM preparation enterprises in the region and proposes improvement paths.

## 2 Methods

### 2.1 Policies analysis based on the PMC index model

#### 2.1.1 Policy collection

Keywords such as “TCM,” “TCM planning,” and “TCM industry” were used to search the official websites of relevant departments, including the state council (SC), National Medical Products Administration (NMPA), National Administration of traditional Chinese medicine (NATCM), Shaanxi provincial People’s government (SPPG), and Shaanxi medical products administration (SMPA), among others, to collect TCMIDPs.

The policy screening strategies were as follows: (1) Policies issued from January 2004 to January 2024 were included; (2) National and Shaanxi provincial policies were incorporated; (3) Policies aimed at TCM enterprises that directly reflect the theme of TCM were considered; (4) Policies that have been modified or repealed were excluded; (5) Policy documents mainly covered regulations plans outlines opinions and notices on TCMIDPs in China. Finally a total of 31 policy texts were collected to discuss the situation of TCMIDPs at the national and Shaanxi provincial levels as shown in Table 1.

**Table 1:** The national and provincial TCMIDPs.

Code	Policy name	Issuing agency	Date issued
Y1	Notice on the Issuance of the “Standards for the Management of Ethical Review of Clinical Research in Traditional Chinese Medicine”	NATCM	2010.09.08
Y2	Notice on the Issuance of the “Specifications for the Construction of Ethical Review Platforms for Clinical Research of Traditional Chinese Medicine” (for Trial Implementation).	NATCM	2011.07.06
Y3	Opinions on Strengthening the Supervision and Management of Traditional Chinese Medicine	NHFPC, NATCM	2016.02.05
……	……	……	……
Y29	Notice on Issuing Several Measures to Accelerate the Characteristic Development of Traditional Chinese Medicine in Shaanxi Province	SPPG	2021.09.17
Y30	“14th Five-Year Plan” for the Development of Health and Wellness in Shaanxi Province	SPHC	2022.04.25
Y31	Notice on Issuing the Three-Year Action Plan for Making Shaanxi a Strong Province in Traditional Chinese Medicine (2024–2026)	SPPG	2024.01.17

NATCM, National Administration of Traditional Chinese Medicine; NHFPC, National Health and Family Planning Commission of the People’s Republic of China; SPPG, Shaanxi Provincial People’s Government; SPHC, Shaanxi Provincial Health Commission.

#### 2.1.2 Construction of the PMC index model

The PMC evaluation index model, initially proposed by Ruiz Estrada (24), was refined using extensive literature on the TCM industry. It included five main steps: (1) preprocessing (filtering and coding) of the TCMIDPs; (2) constructing an evaluation index system for quantitative analysis; (3) categorizing variables and determining parameters; (4) constructing multi-input–output tables; and (5) measuring the PMC index.

Building on the classic framework of the PMC-Index model and the characteristics of TCMIDPs, a comprehensive PMC evaluation index system was developed. Seven primary indicators were included: policy nature (X1), policy timeliness (X2), policy type (X3), policy content (X4), policy evaluation (X5), policy perspective (X6), and policy issuing agency (X7). These primary indicators are further divided into 30 secondary indicators, which aim to cover the content and structural elements of the TCMIDPs comprehensively. Table 2 illustrates the selection and explanations of these evaluation indicators.

**Table 2:** Variable selection and evaluation criteria of PMC-Index model.

Primary indicator	Secondary indicator	Define	Citation
X1 Policy nature	X1-1 prediction	Determine whether the policy involves prediction, suggestion, supervision, guidance, or description; if so, value is 1; otherwise, value is 0.	(19)
	X1-2 suggestion		
	X1-3 supervision		
	X1-4 guidance		
	X1-5 description		
X2 Policy timeliness	X2-1 long-term	Determine whether the policy’s impact period is long-term (> 5 years), medium-term (3–5 years), or short-term (< 3 years); if so, value is 1; otherwise, value is 0.	(22, 36)
	X2-2 medium-term		
	X2-3 short-term		
X3 Policy type	X3-1 funding investment	Determine whether the policy type involves funding investment, talent cultivation, infrastructure construction, etc. if so, value is 1; otherwise, value is 0.	(25, 31, 37)
	X3-2 talent cultivation		
	X3-3 infrastructure construction		
	X3-4 industry shaping		
	X3-5 multidimensional collaboration		
	X3-6 standardization		
	X3-7 international exchange and overseas promotion		
X4 Policy content	X4-1heritage and innovation	Determine whether the policy content includes TCM heritage and innovation, health services, clinical research, technical research, etc. if so, value is 1; otherwise, value is 0.	(26, 38, 39)
	X4-2 health services		
	X4-3clinical research		
	X4-4 technical research		
	X4-5 platform construction		
	X4-6 intellectual property		
	X4-7 cultural dissemination		
X5 Policy evaluation	X5-1 clear responsibilities and authorities	Determine whether the policy has clear responsibilities and authorities, clear objectives, detailed planning, and showcases regional characteristics; if so, value is 1; otherwise, value is 0.	(40)
	X5-2 clear objectives		
	X5-3 detailed planning		
	X5-4 regional characteristics		
X6 Policy perspective	X6-1 macro perspective	Determine whether the policy is formulated at the macro level, meso level, or micro level; if so, value is 1; otherwise, value is 0.	(26)
	X6-2 meso perspective		
	X6-3 micro perspective		
X7 Policy issuing agency	X7-1 SC/NPC	When the policy issuing authority is the State Council/National People’s Congress, other national-level institutions, Shaanxi Provincial People’s Government/Shaanxi Provincial People’s Congress, other provincial-level institutions, the values are 1, 0.9, 0.8, and 0.7, respectively.	(34, 41)
	X7-2 NMPA, NATCM, and other national-level institutions		
	X7-3 SPPG/ SPPC		
	X7-4 SMPA, SPHC, and other provincial-level institutions		

SC, The State Council; NPC, The National People’s Congress; NMPA, National Medical Products Administration; NATCM, National Administration of Traditional Chinese Medicine; SPPG, Shaanxi Provincial People’s Government; SPPC, Shaanxi Provincial People’s Congress.

#### 2.1.3 Measurement of the PMC index

The secondary indicators under the first six primary indicators follow a [0,1] binary distribution (Equations 1 and 2), while the indicators under X7 have specific regulations (Table 2). The values of the seven primary indicators are calculated using Equation 3, and the final PMC index is obtained by summing all the variable values using Equation 4.

The calculation steps for the PMC index model are as follows:


(1) $$X\sim N\left[0,1\right]$$

(2) $$X=\left\{XR:\left[0,1\right]\right\}$$

(3) $$X_{i}=\overset{n}{\underset{j=1}{\sum }}\frac{X_{ij}}{n\left(X_{ij}\right)}$$

(4) $$PMC=\left[\begin{array}{l} X_{1}\left(\overset{5}{\underset{j=1}{\sum }}\frac{X_{1j}}{5}\right)+X_{2}\left(\overset{3}{\underset{j=1}{\sum }}\frac{X_{2j}}{3}\right)\\ +X_{3}\left(\overset{7}{\underset{j=1}{\sum }}\frac{X_{3j}}{7}\right)+X_{4}\left(\overset{3}{\underset{j=1}{\sum }}\frac{X_{4j}}{3}\right)\\ +X_{5}\left(\overset{4}{\underset{j=1}{\sum }}\frac{X_{5j}}{4}\right)+X_{6}\left(\overset{3}{\underset{j=1}{\sum }}\frac{X_{6j}}{3}\right)+X_{7} \end{array}\right]$$

The PMC index results can evaluate the consistency of TCMIDPs. Given that the evaluation index system includes seven primary indicators, the theoretical value of the calculated PMC index ranges from 0 to 7. Based on the PMC index scores of each policy and referring to previous literature (25–27), this study categorizes the policies into four grades: poor (0–1.99), acceptable (2–3.99), good (4–5.99), and excellent (6, 7) (Table 3).

**Table 3:** Policy classification standard based on PMC-Index model.

PMC-Index	0~1.99	2~3.99	4~5.99	6~7
Evaluation	Poor	Acceptable	Good	Excellent

### 2.2 Principal component analysis of TCM industry development in Shaanxi Province

#### 2.2.1 Determination of samples and data sources

Principal component analysis (PCA) (28) is a multivariate statistical method that reduces dimensionality by replacing a larger and more complex set of variables with a smaller set of composite variables, retaining as much information from the original variables as possible. The development of the TCM industry involves extensive information. By applying PCA to this information, the study provides a comprehensive assessment of the development status of TCM production enterprises in Shaanxi Province.

Information was collected from 51 TCM enterprises in Shaanxi Province through questionnaires. The criteria for including enterprises were: (1) the main business involves the production and sales of TCM; (2) the enterprise has complete data on personnel, systems, and finances.

#### 2.2.2 Steps of PCA

Assuming that there are 𝑛 research samples in a comprehensive evaluation, each sample observes 𝑝 evaluation indicators. The steps are as follows: (1) preprocess the raw data to obtain the standardized matrix 𝑍; (2) perform the KMO and Bartlett’s tests; (3) establish the correlation coefficient matrix and determine the number of principal components based on the cumulative contribution rate; (4) linearly combine the principal components and construct a comprehensive evaluation function using the variance contribution rate 𝐴𝑖 of each principal component as weights; (5) calculate the scores for each principal component and the comprehensive evaluation score 𝐹, then comprehensively evaluate all research objects. All calculations were completed using SPSS 26.

Standardized matrix 𝑍:

(5) $$Z_{n\times p}=\left[\begin{array}{ccc} Z_{11} & \cdots & Z_{1p}\\ \vdots & \ddots & \vdots \\ Z_{n1} & \cdots & Z_{np} \end{array}\right]$$

The cumulative contribution rate of the first 𝑘 principal components:

(6) $$M_{k}=\frac{\sum_{i=1}^{k}\lambda_{i}}{\sum_{i=1}^{p}\lambda_{i}},\left(i=1,2,\ldots ,k\right)$$

Expression of the 𝑖-th principal component:


(7) Fi=a1iS1+a2iS2+…+akiSp,i=1,…,k

Comprehensive evaluation function:


(8) $$F=A_{1}F_{1}+A_{2}F_{2}+\ldots +A_{k}F_{k}$$

#### 2.2.3 Empirical analysis

(1) Establishment of the evaluation index system and standards (Table 4)
(2) Preprocess the original data to obtain the standardized matrix 𝑍 (Equation 5).
(3) KMO and Bartlett’s Test.

**Table 4:** Evaluation indicators and standards for TCM industry development.

Primary indicators	Code	Secondary indicators	Code	Secondary indicator calculation
Institutional standard construction	S1	TCM preparation management capacity	S1-1	Score of operating procedures involved in TCM preparation management
		TCM preparation R&D management capacity	S1-2	Score of preparation R&D process and incentives
R&D innovation capability	S2	Number of preparation varieties	S2-1	/
		R&D intensity	S2-2	R&D expenditure / 2022 revenue
Talent team construction	S3	Total number of employees	S3-1	/
		Number of R&D personnel	S3-2	/
		Proportion of employees with bachelor’s degree or higher	S3-3	(Undergraduates + Postgraduates + PhDs) / total number of employees
Enterprise cooperation	S4	Cooperation with other enterprises	S4-1	Assigned value based on original data
Market scale and financial performance	S5	Average total industrial output value over 3 years	S5-1	Average of total industrial output value over 3 years
		Net profit margin	S5-2	2022 net profit / 2022 revenue
		Net profit growth rate	S5-3	2022 net profit / previous year (2021) net profit

PCA was applied to analyze the selected indicators. The results indicated that the KMO values were all above 0.5, and Bartlett’s test of sphericity rejected the null hypothesis of an identity correlation matrix with *p* < 0.001, confirming the suitability for PCA (Supplementary Table S1).

(4) Determine the number of principal components.

Principal components with eigenvalues greater than 1 were extracted, resulting in five principal components that cumulatively explained 78.188% of the variation (Equation 6) (Supplementary Table S2).

(5) Principal component scores (Equations 7 and 8).

The expressions for the five principal components were calculated as follows:


$$\begin{array}{l} F_{1}=0.429S2+0.369S3+0.448S5+0.351S6\\ +0.418S9+0.293S10 \end{array}$$

$$F_{2}=-0.389S1+0.526S4$$

$$F_{3}=0.543S10+0.487S11$$

$$F_{4}=0.724S1–0.593S11$$

$$F_{5}=0.607S4–0.709S8$$

According to the calculation expressions of 𝐹_1_, 𝐹_2_, 𝐹_3_, 𝐹_4_, 𝐹_5_ and the calculation variable function of SPSS, the equation for the comprehensive evaluation of all samples is finally obtained:

Using the expressions for 𝐹_1_, 𝐹_2_, 𝐹_3_, 𝐹_4_, and 𝐹_5_ along with the SPSS calculation variable function, the equation for the comprehensive evaluation of all samples was derived:


$$F=0.403\;F_{1}+0.198\;F_{2}+0.163\;F_{3}+0.120\;F_{4}+0.117\;F_{5}$$

The total evaluation score of all companies (51 companies) was then calculated.

## 3 Results and analysis

### 3.1 Evaluation of TCM industry policies

#### 3.1.1 Overall policy assessment

**Figure 1:**
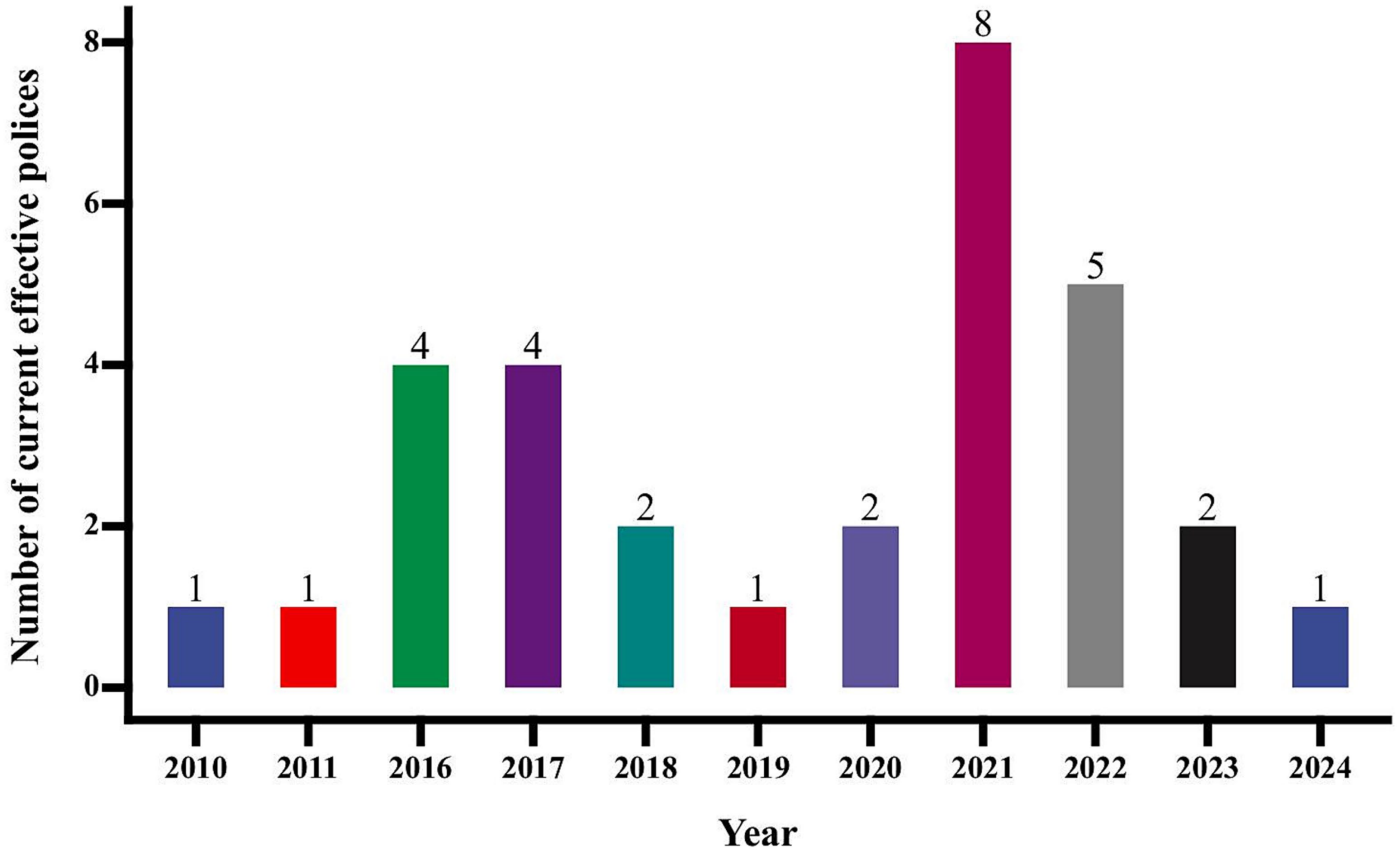
Annual changes in the number of TCMIDPs issued.

According to the trend chart of the annual release of TCMIDPs by the Chinese government (Figure 1), peak periods occurred in 2016 and 2021. The surge in 2016 can be attributed to the enactment of the *Traditional Chinese Medicine Law*, which significantly accelerated the issuance of TCMIDPs, reflecting the government’s increased commitment to advancing the industry (29). Similarly, 2021 marked the launch of *China’s “14th Five-Year Plan,”* during which numerous policy documents were introduced, outlining specific tasks and objectives for TCM development over the plan period.

Table 5 and Supplementary Table S6 show the information of the multi-input/output matrices and PMC indices for 31 TCMIDPs. The average PMC index of the policies is 5.080 ± 1.088, with 58.1% of the TCMIDPs exceeding this value. Policy nature (X1) includes prediction, suggestion, supervision, guidance, and description, while most policies only involve recommendations, guidance, and description, lacking prediction and regulatory aspects. Policy timeliness (X2) includes long-term, medium-term, and short-term development goals. There is still room for improvement in setting long-term development goals in TCM industry policies. In terms of policy type (X3), the lowest average values are observed for funding allocation and standard norms. This suggests that many policies tend to overlook the allocation of financial resources during development and fail to update corresponding standard norms. Policy content (X4) includes inheritance and innovation, health services, clinical research, technological research, platform construction, intellectual property, and cultural dissemination in the development of the TCM industry. Most policies focus more on technological research and pay less attention to cultural dissemination and intellectual property. Additionally, within policy evaluation (X5) and policy perspective (X6), the lowest attention is given to regional characteristics and macro perspectives. Overall, the average values for policy type (X3), policy content (X4), and policy perspective (X6) are lower, being 0.539, 0.622, and 0.656, respectively. This is primarily because several policies serve as standards and norms, covering monotonous policy content, and lacking comprehensive macro-, meso-, and micro-perspectives, thus affecting the PMC-index.

**Table 5:** PMC index and TCMIDPs level.

TCMIDPs	X1	X2	X3	X4	X5	X6	X7	PMC index	Level	Ranking
National policy										
Y1	0.60	1.00	0.14	0.14	0.75	0.33	0.90	3.87	Acceptable	25
Y2	0.60	1.00	0.14	0.29	0.75	0.33	0.90	4.01	Good	24
Y3	0.80	0.33	0.29	0.29	0.75	1.00	0.90	4.35	Good	22
Y4	1.00	1.00	0.86	1.00	1.00	1.00	1.00	6.86	Excellent	1
Y5	0.80	1.00	0.86	0.86	1.00	1.00	0.90	6.41	Excellent	3
……	……	……	……	……	……	……	……	……	……	……
Y20	0.80	0.67	0.43	0.57	1.00	0.67	0.90	5.03	Good	19
Y21	0.60	0.67	0.00	0.43	0.50	0.33	0.90	3.43	Acceptable	29
Y22	1.00	0.33	1.00	1.00	1.00	1.00	0.90	6.23	Excellent	5
Y23	0.60	1.00	0.14	0.00	0.75	0.33	0.90	3.73	Acceptable	27
Provincial policy										
Y24	0.80	1.00	0.86	1.00	0.50	0.67	0.80	5.62	Good	13
Y25	0.60	1.00	0.86	0.71	1.00	0.33	0.80	5.30	Good	15
……	……	……	……	……	……	……	……	……	……	……
Y29	1.00	0.67	0.86	1.00	1.00	0.67	0.80	5.99	Good	7
Y30	1.00	0.67	0.86	0.86	1.00	0.67	0.70	5.75	Good	11
Y31	0.80	0.33	0.71	0.86	1.00	0.67	0.80	5.17	Good	17
National policy average	0.75	0.83	0.50	0.58	0.79	0.70	0.93	5.08	Good	
Provincial policy average	0.80	0.75	0.64	0.73	0.88	0.54	0.75	5.09	Good	
Overall average	0.76	0.81	0.54	0.62	0.81	0.66	0.88	5.08	Good	

According to Table 5, the six policies with excellent consistency are all national policies, ranked from highest to lowest as Y4, Y16, Y5, Y17, Y22, and Y18. These policies are predominantly national development plans or guidelines for the TCM industry, primarily issued by SC or NATCM. Notably, there were no policies with poor consistency. Seven policies exhibited acceptable consistency, characterized by lower PMC indices in policy nature (X1), policy type (X3), policy content (X4), and policy perspective (X6). These policies address only a few aspects of policy nature, are mainly standard norms in policy type, cover fewer aspects in policy content, and emphasize a micro perspective.

#### 3.1.2 Specific policy evaluation

The PMC index for national-level policies ranges from 3.04 to 6.86, with an average of 5.076. For policies of Shaanxi Province, the PMC index ranges from 3.15 to 5.99, with an average of 5.092. Among the 23 national-level TCMIDPs, 6 policies (26.1%) have excellent consistency, 12 (52.2%) are good, and 5 (21.7%) are acceptable. Among the 8 provincial-level TCMIDPs, none are excellent, 6 (75.0%) are good, and 2 (25.0%) are acceptable, with no policies falling into the low consistency category. The quantitative evaluation of policies indicates that among the current effective policies, national-level TCMIDPs have the highest rate of excellent consistency (26.1%) and overall perform well.

**Figure 2:**
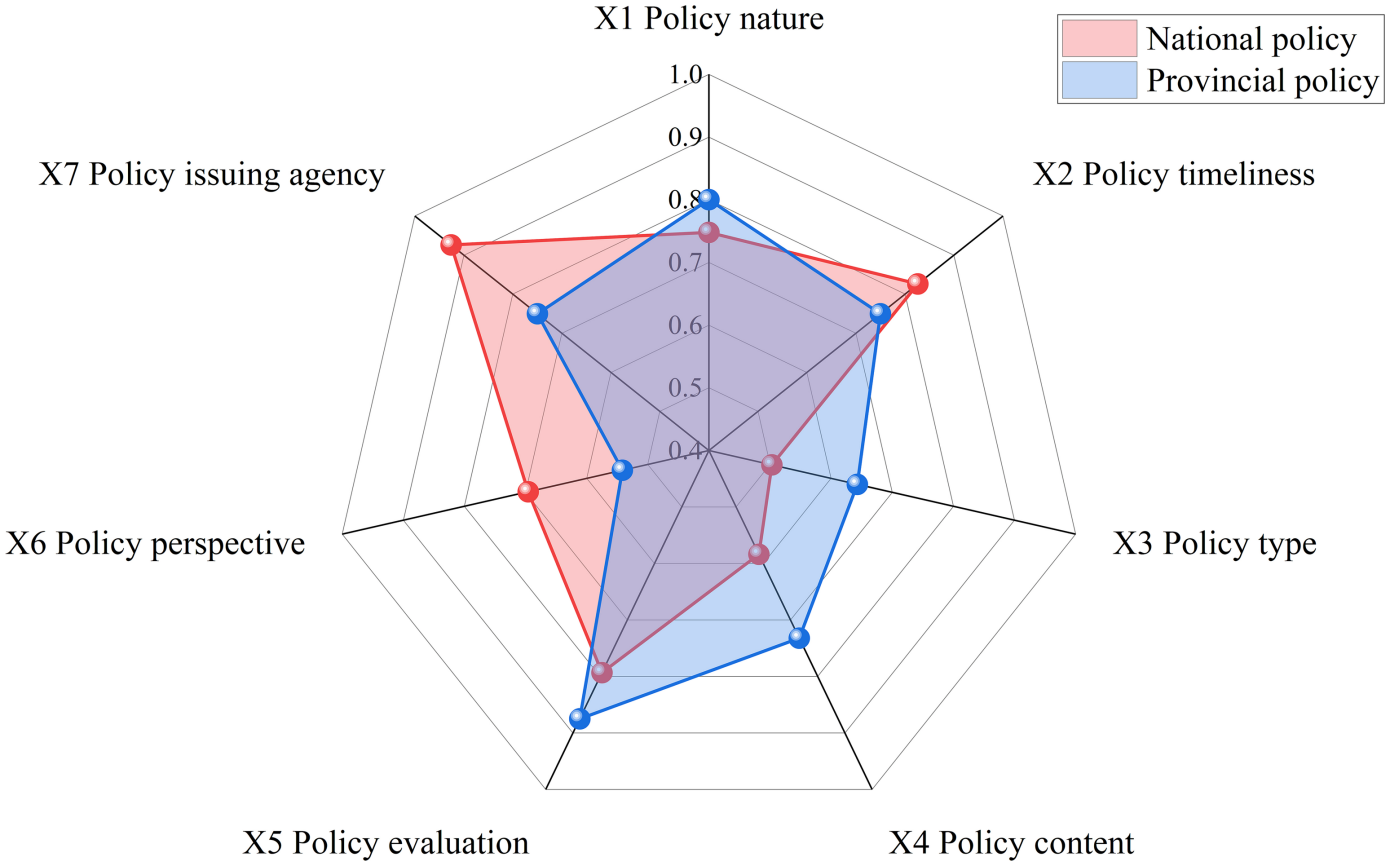
Comparison of primary variables between national and provincial policies.

To clearly display the differences between national and provincial policies, a radar chart was drawn (Figure 2). The national and provincial policies have similar scores for policy nature (X1). In terms of policy timeliness (X2), policy perspective (X6), and issuing agency (X9), national policies score higher than provincial policies. National-level policy issuing agencies have higher levels, and the development goals’ timelines are more comprehensive (22), with thorough planning for long- and short-term development, covering multiple perspectives. This aligns with the objectives of national-level policies, which aim to make plans for nationwide development, requiring higher standards for policy timeliness and development perspectives. Conversely, provincial policies score higher than national policies in policy type (X3), policy content (X4), and policy evaluation (X5). Under the guidance of national-level policies, local governments need to formulate specific policies tailored to local TCM industry development needs (30). Consequently, provincial-level policies are more comprehensive and targeted in terms of policy type and content.

### 3.2 PCA of the TCM industry in Shaanxi Province

#### 3.2.1 Overall situation of the TCM industry

Supplementary Table S4 presents the comprehensive evaluation scores of TCM enterprises in Shaanxi Province. The top three enterprises, based on their comprehensive ranking, are Shaanxi Dongtai Pharmaceutical Company Limited, Xi’an Beilin Pharmaceutical Company Limited, and Shaanxi Hanwang Pharmaceutical Company Limited. They were all established before 2000 and in the cities of Xianyang, Xi’an, and Hanzhong, respectively. Most enterprises are privately owned (90.0%) and concentrate in the Guanzhong area (72.5%), with an average establishment period of 30.35 ± 12.50 years (Table 6).

**Table 6:** Comprehensive evaluation results of TCM enterprises in Shaanxi Province.

Variables	Mean ± SD/ (%)	The top 20 enterprises	The bottom 20 enterprises	*χ^2^*/*t*	*P*
		Mean ± SD/ (%)	Mean ± SD/ (%)		
Enterprise type				1.456	0.605
State-owned	3(7.5%)	2(10.0%)	1(5.0%)		
Private	36(90.0%)	17(85.0%)	19(95.0%)		
Foreign-funded	1 (2.5%)	1(5.0%)	0(0.0%)		
Geographical location				0.435	1.000
Southern Shaanxi	9(22.5%)	4(20.0%)	5(25.0)		
Northern Shaanxi	2(5.0%)	1(5.0%)	1(5.0%)		
Central Shaanxi	29(72.5%)	15(75.0%)	14(70.0%)		
Years of establishment	30.35 ± 12.50	30.95 ± 13.58	31.20 ± 12.33	−0.061	0.952
TCM preparation management capacity	0.99 ± 0.03	1.00 ± 0.01	0.99 ± 0.02	1.042	0.305
TCM preparation R&D management capacity	0.51 ± 0.39	0.81 ± 0.31	0.12 ± 0.09	9.706	<0.001
Number of approved preparation varieties	38.12 ± 40.45	62.45 ± 51.60	18.65 ± 13.26	3.677	0.001
R&D intensity	0.04 ± 0.03	0.037 ± 0.016	0.022 ± 0.022	2.548	0.015
Total number of employees	272.53 ± 283.43	491.25 ± 340.37	103.30 ± 61.60	5.016	<0.001
Number of R&D personnel	18.84 ± 25.18	32.80 ± 34.65	6.65 ± 7.39	3.301	0.003
Proportion of employees with bachelor’s degree or higher	0.22 ± 0.11	0.25 ± 0.09	0.18 ± 0.11	2.292	0.028
Cooperation with other enterprises	1.71 ± 0.50	1.70 ± 0.57	1.60 ± 0.50	0.588	0.560
Average total industrial output value over 3 years	27754.67 ± 45655.73	59799.44 ± 60012.16	5893.58 ± 7840.11	3.983	0.001
Net profit margin	−0.013 ± 0.19	0.10 ± 0.13	−0.11 ± 0.19	4.010	<0.001
Net profit growth rate	0.21 ± 4.97	0.84 ± 1.03	0.68 ± 1.27	0.457	0.651
F1		1.56 ± 1.64	−1.40 ± 0.50	7.716	<0.001
F2		−0.07 ± 0.35	−0.29 ± 0.53	1.603	0.118
F3		0.39 ± 0.45	−0.22 ± 0.56	3.826	<0.001
F4		0.12 ± 0.30	−0.02 ± 0.61	0.862	0.396
F5		0.05 ± 1.02	−0.17 ± 0.77	0.751	0.457
F		0.70 ± 0.62	−0.68 ± 0.35	8.629	<0.001

#### 3.2.2 Analysis of development differences among TCM enterprises

According to Table 6, there are no significant differences (*p* > 0.05) in enterprise type, geographical location, or years of establishment between the top 20 and bottom 20 TCM enterprises in Shaanxi Province. Compared to the bottom 20 enterprises, the top 20 enterprises performed better in several areas: S1-2 (management capability for TCM preparation R&D, 0.81 ± 0.31 vs. 0.12 ± 0.09, *p* < 0.001), S2-1 (number of approved TCM preparation varieties, 62.45 ± 51.60 vs. 18.65 ± 13.26, *p* = 0.001), S2-2 (R&D intensity, 0.037 ± 0.016 vs. 0.022 ± 0.022, *p* = 0.015), S3-1 (total number of employees, 491.25 ± 340.37 vs. 103.30 ± 61.60, *p* < 0.001), S3-2 (number of R&D personnel, 6.65 ± 7.39 vs. 32.80 ± 34.65, *p* = 0.003), S3-3 (proportion of employees with a bachelor’s degree or higher, 0.25 ± 0.09 vs. 0.18 ± 0.11, *p* = 0.028), S5-1 (average total industrial output value over the past 3 years, 59799.44 ± 60012.16 vs. 5893.58 ± 7840.11, *p* = 0.001), and S5-2 (net profit margin, 0.10 ± 0.13 vs. -0.11 ± 0.19, *p* < 0.001), with statistical significance. Conversely, differences in areas such as S1-1 (management capability for TCM preparation configuration), S4-1 (cooperation with other enterprises), and S5-3 (net profit growth rate) are not statistically significant. This suggests that the TCM industry in Shaanxi Province exhibits similar levels of resource allocation and enterprise cooperation. Despite this, the top 20 enterprises continue to hold significant advantages in R&D innovation capability, talent development, and market scale, indicating a trend of steady growth in the TCM industry in recent years.

Based on the comprehensive evaluation score, the differences in the final evaluation scores (𝐹 values, 0.70 ± 0.62 vs. −0.68 ± 0.35, *p* < 0.001) between the top 20 and bottom 20 enterprises are significant. Specifically, it mainly caused by the principal components 𝐹_1_ (1.56 ± 1.64 vs. −1.40 ± 0.50, *p* < 0.001) and 𝐹_3_ (0.39 ± 0.45 vs. -0.22 ± 0.56, *p* < 0.001). According to the principal component matrix (Supplementary Table S3), the variables of S1-2, S2-1, S3-1, S3-2, and S5-1 have larger loadings on the first principal component (F1), and the variable S5-2 has a larger loading on the third principal component (𝐹_3_). This indicates that the main factors contributing to the development imbalance of TCM enterprises in Shaanxi Province are the enterprises’ R&D management capabilities, number of preparation varieties, number of employees and R&D personnel, total industrial output value, and net profit margin.

## 4 Discussion

Based on the analysis of TCMIDPs and PCA results, this study discusses the structure of policies and their impact on the TCM industry from three perspectives.

First, key evaluation dimensions influencing PMC index scores include policy type (X3), policy content (X4), and policy perspective (X6). For X3, low average scores for funding allocation and standardization highlight inadequate financial support and outdated standards crucial to the TCM industry’s development. Financial investment drives innovation and growth in the TCM industry, particularly in fundamental research, technological development, talent cultivation, and industrialization. A lack of funding restricts long-term research and development, hindering technological breakthroughs and large-scale production (31). Similarly, poor standardization undermines the market competitiveness of TCM products. Research indicates that China’s TCM standardization system lacks a comprehensive framework, particularly in raw material sourcing, processing, and healthcare services. These deficiencies undermine industry quality and hinder TCM’s international growth (32). For X4, TCMIDPs show insufficient attention to cultural dissemination and intellectual property (IP) protection, consistent with previous studies (33). Additionally, the absence of comprehensive macro-, meso-, and micro-level perspectives may hinder the coordination of the TCM industry across various stages of development, particularly in areas such as interdisciplinary collaboration and international expansion.

Second, at the policy level, significant differences exist between the content and implementation effectiveness of national and provincial policies. National policies typically offer macro-level guidance, outlining clear objectives, timelines, and a balance between long- and short-term goals (34). In contrast, provincial policies are more localized and tailored, addressing specific regional characteristics and demands. For instance, in Shaanxi Province, the local government leverages regional resources and industry strengths to create comprehensive policies emphasizing practical solutions and short-term goals with high flexibility. However, provincial policies encounter challenges from limited financial and resource capacity, which may compromise their effectiveness (35). A synergistic alignment between national and provincial policies is crucial. National policies should provide overarching directions, while provincial policies should supply detailed implementation strategies to ensure feasibility and practicality.

Finally, based on the current development trends of the traditional Chinese medicine (TCM) industry in Shaanxi Province, it has shown steady growth but has yet to establish a fully integrated industrial cluster. Although progress has been observed in policy support and resource allocation, significant imbalances persist within the industry. Numerous local TCM enterprises remain small in scale, with limited R&D capabilities and low economic efficiency. Key challenges persist in areas such as formulation diversity, technological innovation, standardization, and industrialization, aligning with the findings of the policy analysis. Future policies should prioritize advancing technological innovation and standardization while strengthening support for small- and medium-sized enterprises. Furthermore, integrating and consolidating the industrial chain should be emphasized to enhance collaboration between upstream and downstream enterprises, thereby fostering an innovation-driven industrial cluster. Such measures will strengthen the overall competitiveness of Shaanxi’s TCM industry and promote its high-quality development.

In conclusion, the design and implementation of policies require enhanced precision and specificity. Moving forward, both Shaanxi Province and other regions should focus on detailed implementation measures and ensure the long-term execution of policies. Additionally, promoting coordination and interaction across different policy levels is crucial for achieving the sustainable and balanced development of the TCM industry.

## 5 Conclusion and implications

### 5.1 Conclusion

This study evaluated TCM industry policies and the development status of the TCM industry in Shaanxi Province through the PMC-Index model and PCA, to explore the current improvement paths under policy guidance.

The policy analysis showed that the average PMC index of 31 TCMIDPs is 5.080, with national policies averaging 5.076 and Shaanxi policies averaging 5.092. Among the 23 national TCM policies, 6 (26.1%) were rated excellent in consistency, 12 (52.2%) were good, and 5 (21.7%) were acceptable. Among the 8 provincial TCM policies, none were rated excellent, 6 (75.0%) were good, and 2 (25.0%) were acceptable. The areas needing improvement in TCMIDPs include policy types (X3), policy content (X4), and policy perspectives (X6). National TCM industry policies need improvement in policy types (X3), policy content (X4), and policy evaluation (X5), while Shaanxi’s policies need improvement in policy timeliness (X2), policy perspectives (X6), and issuing institutions (X9).

The TCM industry in Shaanxi Province is mainly concentrated in the Guanzhong area, showing steady growth but has not yet formed a cluster development pattern. Based on PCA, the comprehensive evaluation of the TCM industry extracted five principal components from 11 indicators. The results showed that the top 20 TCM enterprises excelled in R&D management capabilities, the number of preparation varieties, the number of employees and R&D personnel, industrial output value, and net profit margins, indicating an imbalance in the development of TCM enterprises in Shaanxi Province.

Overall, both national and provincial governments have issued a series of policies to drive the growth of TCM industry. Shaanxi’s policies are particularly comprehensive in terms of their types and content, tailored to the current development status of the TCM industry in the province. While based on the PCA results, future policies should focus on the long-term effects of industry development, incorporate more comprehensive perspectives, enhance enterprise R&D capabilities and accelerate pharmaceutical innovation. In terms of policy implementation and content, greater emphasis should be placed to fostering coordination and interaction across different administrative levels, with targeted measures aimed at supporting the comprehensive development of TCM industries.

### 5.2 Implications, limitations, and future work

The study combined the policy perspective with empirical analysis, to explore the development status and improvement paths of the TCM industry in Shaanxi Province. As far as we know, this is the first study to combine TCMIDPs with empirical analysis, providing a new perspective and filling a gap in the literature. Additionally, the results provide empirical evidence for local governments to formulate and improve TCMIDPs, offering valuable references for designing and developing future TCM enterprises.

The study has limitations: (1) The policy text data were sourced from government departments, excluding abolished and invalid policies, which may lead to a partial reflection. (2) Some TCMIDPs were issued in recent years, and their impacts have not fully manifested, requiring longer-term observation and analysis. (3) The PMC-Index model and PCA indicators have room for improvement and optimization. (4) Shaanxi’s TCM industry is relatively small, with insufficient overall development advantages, which may affect the generalizability and representativeness of the results. Therefore, future research should consider the following improvements: (1) Include more provincial policies for regional comparative analysis. (2) Conduct long-term follow-up studies to evaluate the long-term effects of policies and understand their implementation and areas for improvement at different stages. (3) Improve the PMC model and PCA indicators to enhance the scientific and accurate assessment of policy implementation effects.

## Data Availability

The original contributions presented in the study are included in the article/Supplementary material, further inquiries can be directed to the corresponding authors.
