# Supplementary material for: Comprehensive evaluation of the development of traditional Chinese medicine industry in Shaanxi province based on PMC index model
Source: Front Public Health. 2025 Feb 24;13:1500603. doi: 10.3389/fpubh.2025.1500603 (PMC11891234; doi:10.3389/fpubh.2025.1500603)
Supplement: Supplementary file 1 [file Table_1.docx]

**Supplementary files**

Supplementary table 1 KMO and Bartlett’s test for enterprise sample data

| Kaiser-Meyer-Olkin measure of sampling adequacy | Bartlett’s test of sphericity | | |
| --- | --- | --- | --- |
|  | Approx. Chi-Square | Df | Sig. |
| 0.649 | 178.065 | 55 | 0.000 |

Supplementary table 2 The total variance contribution rate of PCA

| Component | Initial eigenvalues | | | Extraction sums of squared loadings | | | | Rotation sums of squared loadings | | |
| --- | --- | --- | --- | --- | --- | --- | --- | --- | --- | --- |
|  | Total | % of variance | Cumulative % | Total | % of variance | | Cumulative % | Total | % of variance | Cumulative % |
| 1 | 3.470 | 31.546 | 31.546 | 3.470 | 31.546 | | 31.546 | 3.069 | 27.900 | 27.900 |
| 2 | 1.699 | 15.447 | 46.992 | 1.699 | 15.447 | | 46.992 | 1.486 | 13.507 | 41.407 |
| 3 | 1.399 | 12.718 | 59.710 | 1.399 | 12.718 | | 59.710 | 1.442 | 13.109 | 54.516 |
| 4 | 1.030 | 9.361 | 69.071 | 1.030 | 9.361 | | 69.071 | 1.370 | 12.453 | 66.969 |
| 5 | 1.003 | 9.117 | 78.188 | 1.003 | 9.117 | | 78.188 | 1.234 | 11.219 | 78.188 |
| 6 | 0.593 | 5.395 | 83.583 |  |  | |  |  |  |  |
| 7 | 0.572 | 5.199 | 88.782 |  |  | |  |  |  |  |
| 8 | 0.481 | 4.638 | 93.150 |  |  | |  |  |  |  |
| 9 | 0.319 | 2.904 | 96.054 |  |  | |  |  |  |  |
| 10 | 0.263 | 2.389 | 98.443 |  |  | |  |  |  |  |
| 11 | 0.171 | 1.557 | 100.000 |  | |  |  |  |  |  |

Supplementary table 3 Principal component matrix

| Secondary indicators | Component | | | | |
| --- | --- | --- | --- | --- | --- |
|  | 1 | 2 | 3 | 4 | 5 |
| TCM preparation management capacity |  | -0.507 |  | 0.735 |  |
| TCM preparation R&D management capacity | 0.799 |  |  |  |  |
| Number of approved preparation varieties | 0.688 |  |  |  |  |
| R&D intensity |  |  |  |  | 0.608 |
| Total number of employees | 0.835 |  |  |  |  |
| Number of R&D personnel | 0.654 |  |  |  |  |
| Proportion of employees with bachelor’s degree or higher |  |  |  |  |  |
| Cooperation with other enterprises |  |  |  |  | -0.710 |
| Average total industrial output value over 3 years | 0.779 |  |  |  |  |
| Net profit margin | 0.546 |  | 0.642 |  |  |
| Net profit growth rate |  |  | 0.576 | -0.602 |  |

Supplementary table 4 Comprehensive evaluation score of TCM enterprises in Shaanxi province

| Enterprise code | Scores of each principal component | | | | | F | Grade |
| --- | --- | --- | --- | --- | --- | --- | --- |
|  | F1 | F2 | F3 | F4 | F5 |  |  |
| 14 | 4.75 | -0.08 | 0.62 | -0.02 | 1.08 | 2.12 | 1 |
| 35 | 4.36 | -0.2 | 0.88 | 0.17 | -0.48 | 1.82 | 2 |
| 19 | 3.79 | -0.61 | 1.32 | 0.12 | -0.95 | 1.52 | 3 |
| 24 | 4.08 | 0.63 | -0.61 | -1.09 | -0.47 | 1.48 | 4 |
| 1 | 3.05 | -0.12 | 0.69 | 0.15 | -0.39 | 1.29 | 5 |
| 44 | 2.1 | 0 | -0.31 | 0.48 | -0.25 | 0.82 | 6 |
| 51 | 1.96 | -0.25 | 0.48 | 0.14 | -0.54 | 0.77 | 7 |
| 13 | 1.34 | 0.17 | 0.79 | 0.17 | -0.06 | 0.71 | 8 |
| 45 | 1.58 | -0.2 | 0.41 | 0.15 | -0.48 | 0.63 | 9 |
| 25 | 1.11 | -0.09 | 0.49 | 0.05 | -0.35 | 0.47 | 10 |
| 31 | 1.33 | -0.7 | 0.14 | 0.22 | -1.05 | 0.32 | 11 |
| 50 | -0.53 | 0.37 | 0.48 | 0.18 | 3.01 | 0.31 | 12 |
| 18 | -0.29 | 0.54 | 0.57 | 0.11 | 1.79 | 0.31 | 13 |
| 42 | 0.73 | -0.19 | 0.49 | 0.13 | -0.47 | 0.3 | 14 |
| 7 | 0.39 | 0.16 | 0.52 | 0.17 | -0.06 | 0.29 | 15 |
| 5 | 0.61 | -0.16 | 0.64 | 0.17 | -0.43 | 0.29 | 16 |
| 17 | 0.54 | -0.63 | 0.36 | 0.13 | 0.44 | 0.22 | 17 |
| 46 | 0.31 | -0.08 | 0.2 | 0.14 | -0.34 | 0.12 | 18 |
| 39 | -0.43 | 0.25 | -0.01 | 0.39 | 1.45 | 0.09 | 19 |
| 11 | 0.4 | -0.16 | -0.31 | 0.34 | -0.44 | 0.07 | 20 |
| 49 | -0.09 | 0.21 | 0.26 | 0.13 | -0.01 | 0.06 | 21 |
| 26 | -0.71 | 1.06 | -0.11 | 0.3 | 0.97 | 0.05 | 22 |
| 38 | -0.04 | -0.18 | 0.8 | 0.1 | -0.46 | 0.04 | 23 |
| 3 | 0.09 | 0.84 | -0.34 | -1 | -0.22 | 0 | 24 |
| 30 | -0.11 | 2.17 | -0.66 | -2.82 | 0.37 | -0.02 | 25 |
| 28 | -1.37 | 1.31 | -0.58 | 0.34 | 2.67 | -0.03 | 26 |
| 2 | -0.87 | 2.59 | -0.01 | -2.71 | 0.86 | -0.06 | 27 |
| 21 | 0.41 | -0.77 | 0.06 | 0.25 | -1.14 | -0.08 | 28 |
| 33 | 0.24 | -0.76 | 0.38 | 0.18 | -1.12 | -0.1 | 29 |
| 34 | -0.13 | 0.54 | -4.34 | 4.16 | 0.37 | -0.11 | 30 |
| 4 | -0.67 | 0.25 | 1.16 | -0.91 | 0.04 | -0.14 | 31 |
| 10 | -0.52 | 0.06 | 0.39 | -0.09 | -0.18 | -0.17 | 32 |
| 9 | -0.39 | -0.18 | 0.3 | 0.2 | -0.45 | -0.17 | 33 |
| 40 | -0.87 | 0.2 | 0.12 | 0.2 | -0.02 | -0.27 | 34 |
| 23 | -0.73 | -0.01 | 0.15 | 0.2 | -0.27 | -0.28 | 35 |
| 8 | -1.3 | 0.2 | -0.11 | 0.16 | 1.39 | -0.32 | 36 |
| 32 | -1.12 | 0.15 | 0.35 | 0.13 | -0.08 | -0.36 | 37 |
| 27 | -0.96 | -0.42 | -0.45 | 0.68 | 0.68 | -0.38 | 38 |
| 43 | -1.13 | -0.19 | 0.02 | 0.42 | -0.46 | -0.49 | 39 |
| 37 | -1.47 | -0.34 | -0.33 | 0.2 | 0.77 | -0.6 | 40 |
| 36 | -1.55 | -0.47 | -0.38 | 0.17 | 0.63 | -0.69 | 41 |
| 16 | -1.31 | -0.86 | 0.71 | 0.15 | -1.24 | -0.71 | 42 |
| 12 | -1.86 | 1.07 | -0.5 | -1.31 | 0.05 | -0.77 | 43 |
| 29 | -1.82 | -0.86 | -0.24 | 0.2 | 0.18 | -0.9 | 44 |
| 48 | -1.52 | -0.86 | -0.11 | 0.26 | -1.24 | -0.91 | 45 |
| 20 | -1.9 | -0.86 | -0.53 | 0.21 | 0.18 | -0.97 | 46 |
| 41 | -1.91 | 0.07 | -0.61 | -1.37 | 0.31 | -0.98 | 47 |
| 47 | -1.78 | -0.86 | -0.48 | 0.42 | -1.24 | -1.06 | 48 |
| 22 | -1.9 | -0.04 | -0.1 | -1.47 | -1.24 | -1.11 | 49 |
| 15 | -1.89 | -0.86 | -0.66 | 0.15 | -1.24 | -1.17 | 50 |
| 6 | -1.95 | -0.86 | -1.99 | 0.18 | 0.18 | -1.24 | 51 |

Supplementary table 5 The national and provincial TCMIDPs

| Code | Policy name | Issuing agency | Date issued |
| --- | --- | --- | --- |
| Y1 | Notice on the Issuance of the “Standards for the Management of Ethical Review of Clinical Research in Traditional Chinese Medicine” | NATCM | 2010.09.08 |
| Y2 | Notice on the Issuance of the “Specifications for the Construction of Ethical Review Platforms for Clinical Research of Traditional Chinese Medicine” (for Trial Implementation) | NATCM | 2011.07.06 |
| Y3 | Opinions on Strengthening the Supervision and Management of Traditional Chinese Medicine | NHFPC, NATCM | 2016.02.05 |
| Y4 | Notice on the Issuance of the “Traditional Chinese Medicine Development Strategic Plan (2016–2030)” | SC | 2016.02.26 |
| Y5 | Notice on the Issuance of “Several Opinions on Accelerating the Construction of the Traditional Chinese Medicine Technology Innovation System” | NATCM | 2016.12.22 |
| Y6 | Traditional Chinese Medicine Law of the People’s Republic of China | NPC | 2016.12.25 |
| Y7 | Notice on Strengthening the Construction of Local Regulations and Systems for Traditional Chinese Medicine | NHFPC, NATCM | 2017.07.10 |
| Y8 | Opinions on Deepening the Reform of the Review and Approval System to Encourage Innovation in Drugs and Medical Devices | SC | 2017.10.08 |
| Y9 | Guiding Opinions on Promoting the Integration of Traditional Chinese Medicine Health Services with the Internet | NATCM | 2017.12.04 |
| Y10 | Guiding Opinions on Strengthening Technological Innovation in Traditional Chinese Medicine Health Services | NATCM, MST | 2018.07.19 |
| Y11 | Notice on the Issuance of the “National Authentic Medicinal Materials Production Base Construction Plan (2018-2025)” | MARA, NMPA, NATCM | 2018.12.18 |
| Y12 | Notice on Carrying out the Construction of Traditional Chinese Medicine Service Export Bases | MC, NATCM | 2019.03.27 |
| Y13 | Notice on Several Policies and Measures to Accelerate the Characteristic Development of Traditional Chinese Medicine | SC | 2021.01.22 |
| Y14 | Notice on Supporting the High-quality Development of National Traditional Chinese Medicine Service Export Bases | MC, NATCM, MFA etc. | 2021.04.29 |
| Y15 | Announcement on Issuing the “Guiding Principles for the Preparation of Traditional Chinese Medicine Theory Application Materials for New Traditional Chinese Medicine Compound Preparations (Trial)” and the “Guiding Principles for the Preparation of Instructions for Traditional Chinese Medicine Compound Preparations of Ancient Classic Formulas (Trial)” | NMPA | 2021.10.15 |
| Y16 | “14th Five-Year Plan” for the Development of the Pharmaceutical Industry | MIIT, NDRC, MST etc. | 2021.12.22 |
| Y17 | Notice on Issuing the “Development Plan for the High-quality Integration of Traditional Chinese Medicine into the Joint Construction of the ‘Belt and Road’ (2021-2025)” | NATCM | 2021.12.31 |
| Y18 | Notice on Issuing the “14th Five-Year Plan” for the Development of Traditional Chinese Medicine | SC | 2022.03.03 |
| Y19 | Notice on Issuing the “14th Five-Year Plan” for Scientific and Technological Innovation in Traditional Chinese Medicine | MST, NATCM | 2022.09.02 |
| Y20 | Notice on Issuing the “14th Five-Year Plan” for the Informatization Development of Traditional Chinese Medicine | NATCM | 2022.11.25 |
| Y21 | Opinions on Strengthening Judicial Protection of Intellectual Property in Traditional Chinese Medicine | SPC | 2022.12.21 |
| Y22 | Notice on Issuing the Implementation Plan for Major Projects in the Revitalization and Development of Traditional Chinese Medicine | SC | 2023.02.10 |
| Y23 | Notice on Issuing the “Administrative Measures for Traditional Chinese Medicine Standards” | NATCM | 2023.10.07 |
| Y24 | Notice on Issuing the “Strategic Plan for the Development of Traditional Chinese Medicine in Shaanxi Province (2017-2030)” | SPPG | 2017.04.18 |
| Y25 | Traditional Chinese Medicine Regulations of Shaanxi Province | SPPC | 2020.04.01 |
| Y26 | Notice on Issuing the “Opinions on Accelerating the Development of the Traditional Chinese Medicine Industry” and the “Traditional Chinese Medicine Industry Development Plan of Shaanxi Province (2020-2030)” | SATCM | 2020.06.06 |
| Y27 | Notice on Issuing the “Measures for the Management of Traditional Chinese Medicine Research Projects in Shaanxi Province (Trial)” | SATCM | 2021.03.18 |
| Y28 | Notice on Issuing the “Detailed Rules for the Administration of Traditional Chinese Medicine Formula Granules in Shaanxi Province (Trial)” | SMPA, SPDIIT, SPHC, etc. | 2021.06.23 |
| Y29 | Notice on Issuing Several Measures to Accelerate the Characteristic Development of Traditional Chinese Medicine in Shaanxi Province | SPPG | 2021.09.17 |
| Y30 | “14th Five-Year Plan” for the Development of Health and Wellness in Shaanxi Province | SPHC | 2022.04.25 |
| Y31 | Notice on Issuing the Three-Year Action Plan for Making Shaanxi a Strong Province in Traditional Chinese Medicine (2024-2026) | SPPG | 2024.01.17 |

National Administration of Traditional Chinese Medicine, NATCM; National Health and Family Planning Commission, NHFPC; The State Council, SC; The National People’s Congress, NPC; Ministry of Science and Technology, MST; Ministry of Agriculture and Rural Affairs, MARA; National Medical Products Administration, NMPA; Ministry of Commerce, MC; Ministry of Foreign Affairs, MFA; Ministry of Industry and Information Technology, MIIT; National Development and Reform Commission, NDRC; The Supreme People’s Court, SPC; Shaanxi Provincial People’s Government, SPPG; Shaanxi Provincial People’s Congress, SPPC; Shaanxi Administration of Traditional Chinese Medicine, SATCM; Shaanxi Medical Products Administration, SMPA; Shaanxi Provincial Department of Industry and Information Technology, SPDIIT; Shaanxi Provincial Health Commission, SPHC.

Supplementary table 6 The multi-input/output matrix of 31 TCMIDPs

| Code | X1(Policy nature) | | | | | X2(Policy timeliness) | | | X3(Policy type) | | | | | | |
| --- | --- | --- | --- | --- | --- | --- | --- | --- | --- | --- | --- | --- | --- | --- | --- |
|  | X1-1 | X1-2 | X1-3 | X1-4 | X1-5 | X2-1 | X2-2 | X2-3 | X3-1 | X3-2 | X3-3 | X3-4 | X3-5 | X3-6 | X3-7 |
| Y1 | 0 | 0 | 1 | 1 | 1 | 1 | 1 | 1 | 0 | 0 | 0 | 0 | 0 | 1 | 0 |
| Y2 | 0 | 0 | 1 | 1 | 1 | 1 | 1 | 1 | 0 | 0 | 0 | 0 | 0 | 1 | 0 |
| Y3 | 0 | 1 | 1 | 1 | 1 | 0 | 0 | 1 | 0 | 0 | 1 | 0 | 0 | 1 | 0 |
| Y4 | 1 | 1 | 1 | 1 | 1 | 1 | 1 | 1 | 0 | 1 | 1 | 1 | 1 | 1 | 1 |
| Y5 | 1 | 1 | 0 | 1 | 1 | 1 | 1 | 1 | 1 | 1 | 1 | 1 | 1 | 0 | 1 |
| Y6 | 0 | 0 | 1 | 1 | 1 | 1 | 1 | 1 | 0 | 1 | 1 | 0 | 0 | 1 | 0 |
| Y7 | 0 | 1 | 0 | 1 | 1 | 0 | 1 | 1 | 0 | 0 | 0 | 0 | 0 | 1 | 0 |
| Y8 | 0 | 1 | 1 | 1 | 1 | 1 | 1 | 1 | 0 | 0 | 0 | 1 | 1 | 0 | 1 |
| Y9 | 1 | 1 | 0 | 1 | 1 | 1 | 1 | 1 | 0 | 1 | 1 | 1 | 1 | 0 | 1 |
| Y10 | 1 | 1 | 0 | 1 | 1 | 1 | 1 | 1 | 1 | 1 | 1 | 1 | 1 | 0 | 1 |
| Y11 | 1 | 1 | 0 | 1 | 1 | 1 | 1 | 1 | 1 | 0 | 1 | 1 | 0 | 0 | 0 |
| Y12 | 1 | 0 | 0 | 1 | 1 | 0 | 1 | 1 | 0 | 0 | 1 | 1 | 0 | 0 | 0 |
| Y13 | 0 | 1 | 0 | 1 | 1 | 1 | 1 | 1 | 1 | 1 | 0 | 1 | 1 | 0 | 0 |
| Y14 | 0 | 1 | 0 | 1 | 1 | 0 | 0 | 1 | 0 | 1 | 1 | 0 | 1 | 0 | 1 |
| Y15 | 0 | 0 | 1 | 1 | 1 | 1 | 1 | 1 | 0 | 0 | 0 | 0 | 0 | 1 | 0 |
| Y16 | 1 | 1 | 1 | 1 | 1 | 1 | 1 | 1 | 1 | 1 | 1 | 1 | 1 | 0 | 1 |
| Y17 | 1 | 1 | 0 | 1 | 1 | 1 | 1 | 1 | 1 | 1 | 1 | 1 | 1 | 0 | 1 |
| Y18 | 1 | 1 | 1 | 1 | 1 | 0 | 1 | 1 | 1 | 1 | 1 | 1 | 1 | 0 | 1 |
| Y19 | 1 | 1 | 0 | 1 | 1 | 0 | 1 | 1 | 0 | 1 | 1 | 1 | 1 | 0 | 0 |
| Y20 | 1 | 1 | 0 | 1 | 1 | 0 | 1 | 1 | 1 | 1 | 1 | 0 | 0 | 0 | 0 |
| Y21 | 0 | 1 | 0 | 1 | 1 | 0 | 1 | 1 | 0 | 0 | 0 | 0 | 0 | 0 | 0 |
| Y22 | 1 | 1 | 1 | 1 | 1 | 0 | 0 | 1 | 1 | 1 | 1 | 1 | 1 | 1 | 1 |
| Y23 | 0 | 0 | 1 | 1 | 1 | 1 | 1 | 1 | 0 | 0 | 0 | 0 | 0 | 1 | 0 |
| Y24 | 1 | 1 | 0 | 1 | 1 | 1 | 1 | 1 | 1 | 1 | 1 | 1 | 1 | 0 | 1 |
| Y25 | 0 | 0 | 1 | 1 | 1 | 1 | 1 | 1 | 0 | 1 | 1 | 1 | 1 | 1 | 1 |
| Y26 | 1 | 1 | 1 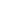 | 1 | 1 | 1 | 1 | 1 | 1 | 0 | 1 | 1 | 1 | 0 | 1 |
| Y27 | 0 | 0 | 1 | 1 | 1 | 1 | 1 | 1 | 0 | 0 | 0 | 0 | 0 | 1 | 0 |
| Y28 | 0 | 0 | 1 | 1 | 1 | 0 | 0 | 1 | 0 | 0 | 0 | 0 | 0 | 1 | 0 |
| Y29 | 1 | 1 | 1 | 1 | 1 | 0 | 1 | 1 | 1 | 1 | 1 | 1 | 1 | 0 | 1 |
| Y30 | 1 | 1 | 1 | 1 | 1 | 0 | 1 | 1 | 1 | 1 | 1 | 1 | 1 | 0 | 1 |
| Y31 | 1 | 1 | 0 | 1 | 1 | 0 | 0 | 1 | 1 | 1 | 1 | 1 | 0 | 0 | 1 |

Supplementary table 6 The multi-input/output matrix of 31 TCMIDPs (Continued)

| Code | X4(Policy content) | | | | | | | X5(Policy evaluation) | | | | X6(Policy perspective) | | | X7(Policy issuing agency) |
| --- | --- | --- | --- | --- | --- | --- | --- | --- | --- | --- | --- | --- | --- | --- | --- |
|  | X4-1 | X4-2 | X4-3 | X4-4 | X4-5 | X4-6 | X4-7 | X5-1 | X5-2 | X5-3 | X5-4 | X6-1 | X6-2 | X6-3 | X7-1 |
| Y1 | 0 | 0 | 1 | 0 | 0 | 0 | 0 | 1 | 1 | 1 | 0 | 0 | 0 | 1 | 0.9 |
| Y2 | 0 | 0 | 1 | 0 | 1 | 0 | 0 | 1 | 1 | 1 | 0 | 0 | 0 | 1 | 0.9 |
| Y3 | 0 | 1 | 0 | 0 | 1 | 0 | 0 | 1 | 1 | 1 | 0 | 1 | 1 | 1 | 0.9 |
| Y4 | 1 | 1 | 1 | 1 | 1 | 1 | 1 | 1 | 1 | 1 | 1 | 1 | 1 | 1 | 1 |
| Y5 | 1 | 0 | 1 | 1 | 1 | 1 | 1 | 1 | 1 | 1 | 1 | 1 | 1 | 1 | 0.9 |
| Y6 | 1 | 1 | 1 | 1 | 0 | 1 | 1 | 1 | 1 | 1 | 0 | 1 | 1 | 1 | 1 |
| Y7 | 1 | 0 | 0 | 0 | 0 | 0 | 0 | 0 | 1 | 0 | 0 | 0 | 1 | 0 | 0.9 |
| Y8 | 1 | 1 | 1 | 1 | 1 | 1 | 0 | 1 | 1 | 1 | 1 | 0 | 1 | 1 | 1 |
| Y9 | 0 | 1 | 0 | 1 | 1 | 0 | 0 | 0 | 1 | 1 | 1 | 0 | 0 | 1 | 0.9 |
| Y10 | 1 | 1 | 1 | 1 | 1 | 0 | 0 | 0 | 0 | 1 | 0 | 1 | 1 | 1 | 0.9 |
| Y11 | 0 | 0 | 0 | 1 | 1 | 0 | 0 | 0 | 1 | 1 | 1 | 1 | 1 | 1 | 0.9 |
| Y12 | 0 | 0 | 0 | 0 | 1 | 0 | 0 | 1 | 1 | 1 | 0 | 0 | 0 | 1 | 0.9 |
| Y13 | 1 | 1 | 1 | 1 | 1 | 1 | 1 | 1 | 1 | 1 | 1 | 0 | 1 | 1 | 1 |
| Y14 | 0 | 1 | 0 | 1 | 1 | 0 | 1 | 1 | 1 | 1 | 1 | 0 | 1 | 1 | 0.9 |
| Y15 | 0 | 0 | 1 | 1 | 0 | 0 | 0 | 1 | 1 | 1 | 1 | 0 | 0 | 1 | 0.9 |
| Y16 | 1 | 1 | 1 | 1 | 1 | 1 | 0 | 1 | 1 | 1 | 1 | 1 | 1 | 1 | 1 |
| Y17 | 1 | 1 | 1 | 1 | 1 | 1 | 1 | 0 | 1 | 1 | 1 | 1 | 1 | 1 | 0.9 |
| Y18 | 1 | 1 | 1 | 1 | 1 | 0 | 1 | 0 | 1 | 1 | 1 | 1 | 1 | 1 | 1 |
| Y19 | 1 | 1 | 1 | 1 | 1 | 0 | 1 | 0 | 1 | 1 | 1 | 0 | 1 | 1 | 0.9 |
| Y20 | 1 | 1 | 0 | 1 | 1 | 0 | 0 | 1 | 1 | 1 | 1 | 0 | 1 | 1 | 0.9 |
| Y21 | 1 | 0 | 0 | 1 | 0 | 1 | 0 | 0 | 1 | 1 | 0 | 0 | 0 | 1 | 0.9 |
| Y22 | 1 | 1 | 1 | 1 | 1 | 1 | 1 | 1 | 1 | 1 | 1 | 1 | 1 | 1 | 0.9 |
| Y23 | 0 | 0 | 0 | 0 | 0 | 0 | 0 | 1 | 1 | 1 | 0 | 0 | 0 | 1 | 0.9 |
| Y24 | 1 | 1 | 1 | 1 | 1 | 1 | 1 | 0 | 1 | 1 | 0 | 0 | 1 | 1 | 0.8 |
| Y25 | 1 | 0 | 1 | 1 | 0 | 1 | 1 | 1 | 1 | 1 | 1 | 0 | 0 | 1 | 0.8 |
| Y26 | 1 | 1 | 0 | 1 | 1 | 1 | 1 | 1 | 1 | 1 | 1 | 0 | 1 | 1 | 0.7 |
| Y27 | 0 | 0 | 1 | 1 | 0 | 0 | 0 | 1 | 1 | 1 | 0 | 0 | 0 | 1 | 0.7 |
| Y28 | 0 | 0 | 1 | 1 | 0 | 0 | 0 | 1 | 1 | 1 | 0 | 0 | 0 | 1 | 0.7 |
| Y29 | 1 | 1 | 1 | 1 | 1 | 1 | 1 | 1 | 1 | 1 | 1 | 0 | 1 | 1 | 0.8 |
| Y30 | 1 | 1 | 1 | 1 | 1 | 0 | 1 | 1 | 1 | 1 | 1 | 0 | 1 | 1 | 0.7 |
| Y31 | 1 | 1 | 0 | 1 | 1 | 1 | 1 | 1 | 1 | 1 | 1 | 0 | 1 | 1 | 0.8 |

Supplementary table 7 PMC index and TCMIDPs level

| TCMIDPs | X1 | X2 | X3 | X4 | X5 | X6 | X7 | PMC index | Level | Ranking |
| --- | --- | --- | --- | --- | --- | --- | --- | --- | --- | --- |
| National policy |  |  |  |  |  |  |  |  |  |  |
| Y1 | 0.60 | 1.00 | 0.14 | 0.14 | 0.75 | 0.33 | 0.90 | 3.87 | Acceptable | 25 |
| Y2 | 0.60 | 1.00 | 0.14 | 0.29 | 0.75 | 0.33 | 0.90 | 4.01 | Good | 24 |
| Y3 | 0.80 | 0.33 | 0.29 | 0.29 | 0.75 | 1.00 | 0.90 | 4.35 | Good | 22 |
| Y4 | 1.00 | 1.00 | 0.86 | 1.00 | 1.00 | 1.00 | 1.00 | 6.86 | Excellent | 1 |
| Y5 | 0.80 | 1.00 | 0.86 | 0.86 | 1.00 | 1.00 | 0.90 | 6.41 | Excellent | 3 |
| Y6 | 0.60 | 1.00 | 0.43 | 0.86 | 0.75 | 1.00 | 1.00 | 5.64 | Good | 12 |
| Y7 | 0.60 | 0.67 | 0.14 | 0.14 | 0.25 | 0.33 | 0.90 | 3.04 | Acceptable | 31 |
| Y8 | 0.80 | 1.00 | 0.43 | 0.86 | 1.00 | 0.67 | 1.00 | 5.75 | Good | 10 |
| Y9 | 0.80 | 1.00 | 0.71 | 0.43 | 0.75 | 0.33 | 0.90 | 4.93 | Good | 20 |
| Y10 | 0.80 | 1.00 | 0.86 | 0.71 | 0.25 | 1.00 | 0.90 | 5.52 | Good | 14 |
| Y11 | 0.80 | 1.00 | 0.43 | 0.29 | 0.75 | 1.00 | 0.90 | 5.16 | Good | 18 |
| Y12 | 0.60 | 0.67 | 0.29 | 0.14 | 0.75 | 0.33 | 0.90 | 3.68 | Acceptable | 28 |
| Y13 | 0.60 | 1.00 | 0.57 | 1.00 | 1.00 | 0.67 | 1.00 | 5.84 | Good | 9 |
| Y14 | 0.60 | 0.33 | 0.57 | 0.57 | 1.00 | 0.67 | 0.90 | 4.64 | Good | 21 |
| Y15 | 0.60 | 1.00 | 0.14 | 0.29 | 1.00 | 0.33 | 0.90 | 4.26 | Good | 23 |
| Y16 | 1.00 | 1.00 | 0.86 | 0.86 | 1.00 | 1.00 | 1.00 | 6.71 | Excellent | 2 |
| Y17 | 0.80 | 1.00 | 0.86 | 1.00 | 0.75 | 1.00 | 0.90 | 6.31 | Excellent | 4 |
| Y18 | 1.00 | 0.67 | 0.86 | 0.86 | 0.75 | 1.00 | 1.00 | 6.13 | Excellent | 6 |
| Y19 | 0.80 | 0.67 | 0.57 | 0.86 | 0.75 | 0.67 | 0.90 | 5.21 | Good | 16 |
| Y20 | 0.80 | 0.67 | 0.43 | 0.57 | 1.00 | 0.67 | 0.90 | 5.03 | Good | 19 |
| Y21 | 0.60 | 0.67 | 0.00 | 0.43 | 0.50 | 0.33 | 0.90 | 3.43 | Acceptable | 29 |
| Y22 | 1.00 | 0.33 | 1.00 | 1.00 | 1.00 | 1.00 | 0.90 | 6.23 | Excellent | 5 |
| Y23 | 0.60 | 1.00 | 0.14 | 0.00 | 0.75 | 0.33 | 0.90 | 3.73 | Acceptable | 27 |
| Provincial policy |  |  |  |  |  |  |  |  |  |  |
| Y24 | 0.80 | 1.00 | 0.86 | 1.00 | 0.50 | 0.67 | 0.80 | 5.62 | Good | 13 |
| Y25 | 0.60 | 1.00 | 0.86 | 0.71 | 1.00 | 0.33 | 0.80 | 5.30 | Good | 15 |
| Y26 | 1.00 | 1.00 | 0.71 | 0.86 | 1.00 | 0.67 | 0.70 | 5.94 | Good | 8 |
| Y27 | 0.60 | 1.00 | 0.14 | 0.29 | 0.75 | 0.33 | 0.70 | 3.81 | Acceptable | 26 |
| Y28 | 0.60 | 0.33 | 0.14 | 0.29 | 0.75 | 0.33 | 0.70 | 3.15 | Acceptable | 30 |
| Y29 | 1.00 | 0.67 | 0.86 | 1.00 | 1.00 | 0.67 | 0.80 | 5.99 | Good | 7 |
| Y30 | 1.00 | 0.67 | 0.86 | 0.86 | 1.00 | 0.67 | 0.70 | 5.75 | Good | 11 |
| Y31 | 0.80 | 0.33 | 0.71 | 0.86 | 1.00 | 0.67 | 0.80 | 5.17 | Good | 17 |
| National policy average | 0.75 | 0.83 | 0.50 | 0.58 | 0.79 | 0.70 | 0.93 | 5.08 | Good |  |
| Provincial policy average | 0.80 | 0.75 | 0.64 | 0.73 | 0.88 | 0.54 | 0.75 | 5.09 | Good |  |
| Overall average | 0.76 | 0.81 | 0.54 | 0.62 | 0.81 | 0.66 | 0.88 | 5.08 | Good |  |
